# 基于SiO_2_@Fe_3_O_4_的磁性纳米材料分离富集谷物中痕量黄曲霉毒素B_1_

**DOI:** 10.3724/SP.J.1123.2022.03002

**Published:** 2022-08-08

**Authors:** Xiaohan LI, Yingying LU, Yongzhen DONG, Feng JIANG, Zhiyong FAN, Hui PAN, Mingjun LIU, Yiping CHEN

**Affiliations:** 1.华中农业大学食品科学技术学院, 湖北 武汉 430070; 1. College of Food Science and Technology, Huazhong Agricultural University, Wuhan 430070, China; 2.湖北省食品质量安全监督检验研究院, 湖北 武汉 430071; 2. Hubei Provincial Institute for Food Supervision and Test, Wuhan 430071, China; 3.荆州市食品药品检验所, 湖北 荆州 434000; 3. Jingzhou Institute for Food and Drug Control, Jingzhou 434000, China

**Keywords:** 高效液相色谱-串联质谱, 磁性纳米粒子, 黄曲霉毒素B_1_, 谷物, 抗体, high performance liquid chromatography-tandem mass spectrometry (HPLC-MS/MS), magnetic nanoparticles, aflatoxin B_1_ (AFB_1_), grain, antibody (Ab)

## Abstract

将SiO_2_包覆的Fe_3_O_4_磁性纳米材料(SiO_2_@Fe_3_O_4_)表面偶联识别黄曲霉毒素B_1_(AFB_1_)的抗体(Ab),用于特异性分离富集谷物中的AFB_1_,进而与高效液相色谱-串联质谱法(HPLC-MS/MS)结合,用于大米、玉米和小麦中AFB_1_的高效准确检测。采用微波辅助水热合成法制备得到Fe_3_O_4_磁性纳米颗粒,并用100 μL正硅酸乙酯(TEOS)对其进行SiO_2_的包覆,得到SiO_2_@Fe_3_O_4_磁性纳米材料,随后进行抗体的偶联得到Ab-SiO_2_@Fe_3_O_4_;以pH=7.4的磷酸盐缓冲液(PBS)作为富集缓冲液,加入8 mg Ab-SiO_2_@Fe_3_O_4_,在37 ℃下反应10 min进行AFB_1_的分离富集,随后采用甘氨酸-盐酸(Gly-HCl)缓冲液对Ab-SiO_2_@Fe_3_O_4_分离富集的AFB_1_进行洗涤,将洗涤液氮吹后复溶,采用高效液相色谱-串联质谱法检测。在最佳条件下,方法检测AFB_1_的线性范围为2~50 μg/L,相关系数(*R*^2^)>0.99,检出限为0.04 μg/kg,定量限为0.13 μg/kg。在4个不同加标水平下,AFB_1_在3种谷物基质中的加标回收率为76.21%~92.85%, RSD≤5.29%。大米、玉米和小麦等实际谷物样品中AFB_1_的测定结果显示,在1个小麦样品和2个玉米样品中检出AFB_1_,其含量分别为0.38、0.13和0.47 μg/kg,其他样品中并未发现AFB_1_。方法将磁性纳米材料与HPLC-MS/MS相结合,实现了AFB_1_的高效分离富集,富集材料成本低廉,储存性能好,在30 min内即可完成前处理过程,可在较短的时间内实现大批量样品的实际分析,在谷物中真菌毒素的检测方面具有良好的应用前景。

## Abstract (en)

In this study, a magnetic nanomaterial antibody (Ab)-SiO_2_@Fe_3_O_4_ was synthesized, which was employed to absorb aflatoxin B_1_ (AFB_1_) in complicated grain matrices. The Ab-SiO_2_@Fe_3_O_4_ material was then paired with high performance liquid chromatography-tandem mass spectrometry (HPLC-MS/MS) for subsequent accurate detection. The Ab-SiO_2_@Fe_3_O_4_ material has a specific adsorption capacity for AFB_1_ because of the stable and specific biological binding between antigen and antibody. This process can achieve the identification between the material and food matrix quickly, thereby completing the separation and enrichment process. Then, high sensitivity and high accuracy HPLC-MS/MS were employed for signal readout and actual quantification, which can significantly increase the detection efficiency and enable high-throughput detection of numerous samples. In the pretreatment process, Fe_3_O_4_ was first synthesized by microwave-assisted hydrothermal synthesis within 1 h, and Ab-SiO_2_@Fe_3_O_4_ was then produced using the enhanced Stober’s approach. This material with high adsorption performance was synthesized under relatively mild conditions and short time. To obtain Ab-SiO_2_@Fe_3_O_4_ materials with uniform particle size, magnetic properties, and dispersibility that met the requirements, synthesis conditions of Ab-SiO_2_@Fe_3_O_4_ and conditions for capturing the AFB_1_ target were analyzed. The findings demonstrated that the best effect was obtained when the dosage of FeCl_3_·6H_2_O was 10.0 mmol, the heating time was 40 min, and 100 μL tetraethoxysilane was employed for SiO_2_ coating. The AFB_1_ antibody was then combined with the surface of SiO_2_@Fe_3_O_4_ under several conditions. The findings revealed that the best coupling efficiency of Ab could be obtained when the concentration of 2-morpholinoethanesulfonic acid monohydrate (MES) was 10 mmol/L, pH was 6.5, and the molar ratio of 1-(3-dimethylaminopropyl)-3-ethylcarbodiimide hydrochloride (EDC)∶*N*-hydroxysuccinimide substances (NHS) was 2∶1. The coupling buffer was then selected as phosphate buffer (PBS) with pH=7.4, and 8 mg Ab-SiO_2_@Fe_3_O_4_ was employed to separate and enrich AFB_1_ at 37 ℃ for 10 min. In the actual detection, acetonitrile-water-formic acid (85∶10∶5, v/v/v) was employed as the extraction solution. After ultrasonic extraction for 10 min, Ab-SiO_2_@Fe_3_O_4_ was employed to separate and enrich AFB_1_ in the extract. The supernatant was dried with nitrogen and reconstituted with 1-mL acetonitrile. The solution was then filtered through a 0.22 μm filter and detected using HPLC-MS/MS, thereby realizing the quick and quantitative detection of AFB_1_. AFB_1_ had an excellent linear relationship in the range of 2-50 μg/L under the optimal analytical conditions, and the correlation coefficient was less than 0.99. The LOD was 0.04 μg/kg, and the LOQ was 0.13 μg/kg. The spiked recoveries of AFB_1_ in three grain matrices ranged from 76.21% to 92.85% with RSD≤5.29% at four different spiked levels. The approach was applied to the determination and analysis of AFB_1_ in 30 real grain samples of rice, corn, and wheat. The findings demonstrated that AFB_1_ was detected in one wheat sample and two corn samples, and its content was 0.38, 0.13, and 0.47 μg/kg, respectively, and no toxins were found in other samples. The approach combined Ab-SiO_2_@Fe_3_O_4_ magnetic nanomaterials with HPLC-MS/MS, which could obtain high-efficiency separation and enrichment of AFB_1_. Furthermore, the low-cost Ab-SiO_2_@Fe_3_O_4_ could be stored for more than a week and complete the pretreatment process within 30 min. This effective pretreatment process combined with HPLC-MS/MS could realize the analysis of several samples within a short time, and had a promising application prospect in the detection of AFB_1_ in grains.

谷物在生长、储存和运输过程中容易受到真菌毒素污染。黄曲霉毒素作为真菌毒素的代表种类之一,是迄今为止发现的最强致癌物^[1]^,其中黄曲霉毒素B_1_(AFB_1_)为Ⅰ类致癌物,毒性极强,即使在低水平下也可能导致肝脏受损,从而诱发癌症,危及生命。由于AFB_1_的明显毒性效应,GB 2761-2017《食品安全国家标准 食品中真菌毒素限量》明确了粮油谷物中黄曲霉毒素的限量,规定玉米、大米和小麦中AFB_1_分别不得超过20、10和5 μg/kg^[2]^。因此,即使在谷物样品中以极微量存在的AFB_1_,其毒性也不容忽视。且谷物中含有较多的淀粉和脂肪,这无疑增加了样品提取富集的难度^[3]^,对样品中的痕量AFB_1_进行充分、快速提取,对检测准确性和检测效率至关重要^[4]^。因此建立一种基于高效前处理技术的准确分析方法对谷物中痕量AFB_1_的检测具有重要意义。

目前,谷物中AFB_1_的检测方法主要是薄层色谱法、酶联免疫吸附法(ELISA)、高效液相色谱法(HPLC)和高效液相色谱-串联质谱法(HPLC-MS/MS)等^[5,6]^。其中,薄层色谱法的灵敏度较低,实验操作较为繁琐且重复性较差^[7]^;酶联免疫吸附法需要重复洗涤,耗时较长^[8]^; HPLC多采用紫外检测器,灵敏度较低,而HPLC-MS/MS将质谱作为检测器,适用性非常广泛,其同时具备质谱的高灵敏度与HPLC的高分离性,能够实现AFB_1_的高效、准确、灵敏的定量定性检测,从而弥补HPLC技术的不足^[9]^。郭芳芳等^[10]^采用液相色谱-串联质谱法对小麦粉中AFB_1_进行检测,结果显示线性良好,相关系数(*R*^2^)大于0.999,定量限为0.1 μg/kg,且加标回收率可达90.3%。目前对于黄曲霉前处理方法的研究主要集中在固相萃取小柱和免疫亲和柱上,而对于磁性纳米材料的研究则较少。赵颖等^[11]^采用核酸适配体亲和柱进行净化,其柱容量可达(334.6±18.2) ng,随后结合HPLC对莲子中的AFB_1_进行定量分析;Zhao等^[12]^采用多毒素复合免疫亲和柱,结合HPLC-MS/MS实现了中药中6种真菌毒素的同时检测,检出限(LOD)为7 pg/mL,定量限(LOQ)为20 pg/mL。虽然免疫亲和柱能够较好地实现痕量AFB_1_的提取效果,但其成本很高,因此针对谷物中的痕量AFB_1_亟须开发一种简单、快速、低成本的前处理方法^[13]^。本方法合成了一种抗体(Ab)-SiO_2_@Fe_3_O_4_磁性纳米材料,其偶联的AFB_1_抗体可以定向捕获谷物样品中的AFB_1_,磁分离后可实现目标分析物AFB_1_与样品复杂基质的快速分离,大大提高了前处理效率。

基于此,本研究利用Ab-SiO_2_@Fe_3_O_4_磁性纳米材料对谷物样品中的AFB_1_进行免疫磁分离富集,将该材料捕获的AFB_1_洗涤下来,结合HPLC-MS/MS对大米、玉米、小麦3种谷物样品中的AFB_1_进行高效准确检测。该磁性纳米材料具有合成方法简单、与复杂基质分离快速的优点,且基于该材料分离富集的前处理过程操作简单、方便、无需多次洗脱,为谷物中痕量黄曲霉毒素的快速分离富集和准确测定提供了简单高效的手段。

## 1 实验部分

### 1.1 仪器、试剂与材料

SiO_2_@Fe_3_O_4_磁性纳米材料的形貌通过Zeiss Gemini300透射电子显微镜(TEM)进行表征,粒径通过Nano ZS动态光散射(DLS)进行表征,化学成分通过IS50傅里叶变换红外光谱仪(FT-IR)进行表征。SuperMag磁性分离架购自美国Ocean NanoTech公司,MKX-H2C1微波合成仪购自青岛迈可威微波应用技术有限公司。Xevo TQ-S液相色谱-串联质谱联用仪、0.22 μm有机滤膜购自美国Waters公司,实验用水为Milli-Q型超纯水,超纯水机购自美国Millipore公司。MX-S可调式混匀仪购自南京润耀生物科技有限公司,BX超声波清洗机购自上海新苗医疗器械制造有限公司,NDK200-2N型氮吹仪购自杭州米欧仪器有限公司。

AFB_1_、赭曲霉毒素A(OTA)、脱氧雪腐镰刀菌烯醇(DON)、玉米赤霉烯酮(ZEN)标准品购自上海源叶生物科技有限公司,纯度均大于98%; Ab(2 mg/mL)由华中农业大学国家兽药残留实验室提供。(3-氨基丙基)三乙氧基硅烷(APTES)、正硅酸乙酯(TEOS)均购自上海阿拉丁生化科技股份有限公司。甲醇、乙腈、甲酸均为色谱纯,购自德国默克公司。使用的所有其他化学品和试剂均为分析纯。大米、玉米和小麦3种谷物样本均从当地市场购买并密封,置于-20 ℃保存使用。

### 1.2 Ab-SiO_2_@Fe_3_O_4_的合成

#### 1.2.1 Fe_3_O_4_磁性纳米颗粒的合成

采用微波辅助水热法^[14]^合成Fe_3_O_4_磁性纳米颗粒,将10.0 mmol六水合氯化铁(FeCl_3_·6H_2_O)、4.0 mmol三水合醋酸钠(NaAc·3H_2_O)和3.0 mmol十二烷基磺酸钠(SDS)溶解在25 mL乙二醇中。搅拌后将混合物转移到50 mL微波合成反应容器中,设置微波合成仪的加热参数。第一加热段为150 ℃下加热20 min,第二加热段为200 ℃下加热40 min,冷却至室温,用10 mL去离子水和无水乙醇交替洗至中性,真空干燥,在常温下保存。

#### 1.2.2 SiO_2_@Fe_3_O_4_的合成

为了增加Fe_3_O_4_磁性纳米颗粒的亲水性,利于后续基团修饰,采用聚乙烯吡咯烷酮(PVP)对Fe_3_O_4_磁性纳米颗粒的性状进行改良,随后进行二氧化硅的包覆^[9,15]^。取10 mg Fe_3_O_4_磁性纳米颗粒与1 g PVP在20 mL纯水中反应1 h,用75%乙醇洗涤。加入1 mL氨水、100 μL TEOS,用乙醇稀释到10 mL,搅拌30 min。随后进行磁分离、干燥、研磨,在常温下保存。

#### 1.2.3 Ab-SiO_2_@Fe_3_O_4_的制备

在上一步制得的SiO_2_@Fe_3_O_4_磁性纳米材料中加入10%(v/v)APTES水溶液使其氨基化,随后进行抗体的偶联。在SiO_2_@Fe_3_O_4_磁性纳米材料中加入40 μL 1-(3-二甲氨基丙基)-3-乙基碳二亚胺盐酸盐(EDC, 5 mg/mL)、20 μL *N*-羟基琥珀酰亚胺(NHS, 5 mg/mL)和440 μL 2-(*N*-吗啡啉)乙磺酸(MES)溶液(10 mol/L, pH=6.5),混匀后加入100 μg AFB_1_抗体,在37 ℃下翻转混匀60 min。最后用磷酸盐缓冲液(PBS, 0.05 mol/L, pH=7.4)洗涤除去未结合的抗体,于4 ℃下保存,待使用。

### 1.3 谷物中AFB_1_的提取

将10 mL乙腈-水-甲酸(85∶10∶5, v/v/v)溶液加入大米、玉米、小麦3份谷物样品(均为5 g)中涡旋混匀,超声提取10 min, 7000 r/min下离心5 min,取上清液氮吹后采用10 mL PBS(pH=7.4)复溶,于4 ℃下储存。

### 1.4 Ab-SiO_2_@Fe_3_O_4_的分离富集

向1.3节得到的10 mL提取液中加入8 mg Ab-SiO_2_@Fe_3_O_4_磁性纳米材料,在37 ℃下涡旋混匀10 min,磁分离后弃去上清液,采用1 mL甘氨酸-盐酸(Gly-HCl)缓冲液(0.1 mol/L,含2% Tween-20)^[16]^对磁分离后剩余的Ab-SiO_2_@Fe_3_O_4_磁性纳米材料进行洗脱,将其捕获的AFB_1_洗涤下来。随后再次磁分离,将上清液氮吹至近干,用1 mL乙腈复溶,过0.22 μm滤膜待进样检测。

### 1.5 Ab-SiO_2_@Fe_3_O_4_的富集能力计算

Ab-SiO_2_@Fe_3_O_4_磁性纳米材料对AFB_1_的捕获能力由下式计算:


(1) $Q_e=\frac{C_0-Ce}{m}\times V$

式中,*Q*_e_为Ab-SiO_2_@Fe_3_O_4_磁性纳米材料达到富集平衡时的捕获能力(mg/g), *C*_0_为溶液中AFB_1_的初始质量浓度(μg/L), *C*_e_为达到富集平衡时溶液中剩余AFB_1_的质量浓度(μg/L), *m*为添加的Ab-SiO_2_@Fe_3_O_4_磁性纳米材料的质量(mg), *V*为AFB_1_溶液的体积(mL)。

### 1.6 高效液相色谱-串联质谱条件

色谱条件:色谱柱为ACQUITY BEH C18色谱柱(50 mm×2.1 mm, 1.7 μm);柱温为35 ℃;进样量为2 μL,流速为0.3 mL/min;流动相为含0.1%甲酸的乙腈,等度洗脱,所有流动相在使用前通过0.22 μm滤膜过滤,并在超声中脱气。

质谱条件:离子源为电喷雾电离源;质谱扫描方式为多反应监测(MRM)模式;喷雾电压为4.5 kV,喷雾电流为8 μA;鞘气N_2_(99.99%),流量35 L/min;辅助气体N_2_(99.99%),流量5 L/min;离子传输毛细管温度300 ℃;毛细管电压16 V。

## 2 结果与讨论

### 2.1 Ab-SiO_2_@Fe_3_O_4_合成条件的优化

#### 2.1.1 SiO_2_包覆条件优化

为了合成性能优异的Fe_3_O_4_磁性纳米颗粒,采用3组单因素实验对FeCl_3_·6H_2_O的用量(温度为200 ℃,时间为40 min)以及主加热阶段(第二加热阶段)的时间(温度为200 ℃, FeCl_3_·6H_2_O的用量为10.0 mmol)和温度(FeCl_3_·6H_2_O的用量为10.0 mmol,时间为40 min)进行了优化。采用DLS比较不同条件下合成的Fe_3_O_4_磁性纳米颗粒的粒径分布。

结果表明,Fe_3_O_4_的粒径随着FeCl_3_·6H_2_O用量和加热时间的增加而增加,这与之前的研究结果相似^[17]^,说明微波辅助水热合成法和经典水热方法具有相同的促反应机理,并且当FeCl_3_·6H_2_O的用量为10.0 mmol(见图1a)、第二加热阶段的加热时间为40 min(见图1b)时具有相对均匀的粒径分布,峰宽较窄。而Fe_3_O_4_磁性纳米颗粒的粒径随着第二加热阶段加热温度的增加出现先增加后减小的趋势,这是由于较高的反应温度能够提高反应速率,加快反应的进行,从而加速晶核的形成和生长,但反应温度过高,会导致一些粒径较大的磁性纳米粒子破裂或变为空心状态,从而导致产物不纯,粒径不均(见图1c)。因此选择200 ℃作为合成温度,在这一条件下制备的Fe_3_O_4_磁性纳米颗粒粒径分布均匀、分散性好,有利于后续SiO_2_的表面修饰,实现更加有效的分离富集过程。

**图1:**
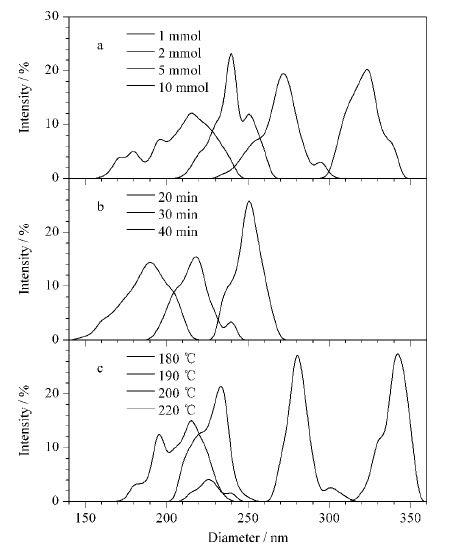
(a)FeCl_3_·6H_2_O用量、(b)时间和(c)主加热阶段温度 对Fe_3_O_4_磁性纳米颗粒粒径分布的影响

以Fe_3_O_4_磁性纳米颗粒为原料进一步合成了SiO_2_@Fe_3_O_4_磁性纳米材料,其中SiO_2_包覆层的厚度对材料的磁性、分散性都有一定的影响。我们采用动态光散射法得出不同TEOS溶液用量(60、80、100、120 μL)下合成的SiO_2_@Fe_3_O_4_的粒径分布曲线,从而确定SiO_2_包覆层的厚度和材料均匀性;用去离子水将一定质量的SiO_2_@Fe_3_O_4_配制成0.01 mg/mL的溶液,取10 mL SiO_2_@Fe_3_O_4_溶液于量筒中,采用溶液中的SiO_2_@Fe_3_O_4_磁性纳米材料在量筒底部外加磁场的存在下完全沉降到底部所需的时间作为磁分离时间,用来反映磁性纳米材料的磁性能。磁分离时间越短,说明磁性纳米材料的磁性能就越强。由图2可知,随着TEOS用量的增加磁颗粒的沉降时间逐渐增加,主要是因为TEOS的用量与SiO_2_包覆层厚度成正相关,从而影响磁颗粒的沉降时间。当采用TEOS的用量为100 μL时,粒径分布均匀,峰宽最窄,47.18%的SiO_2_@Fe_3_O_4_磁性纳米材料的粒径分布在270~290 nm之间,且在4 s内就可以完成磁分离,具有较好的均匀性和磁性能。

**图 2:**
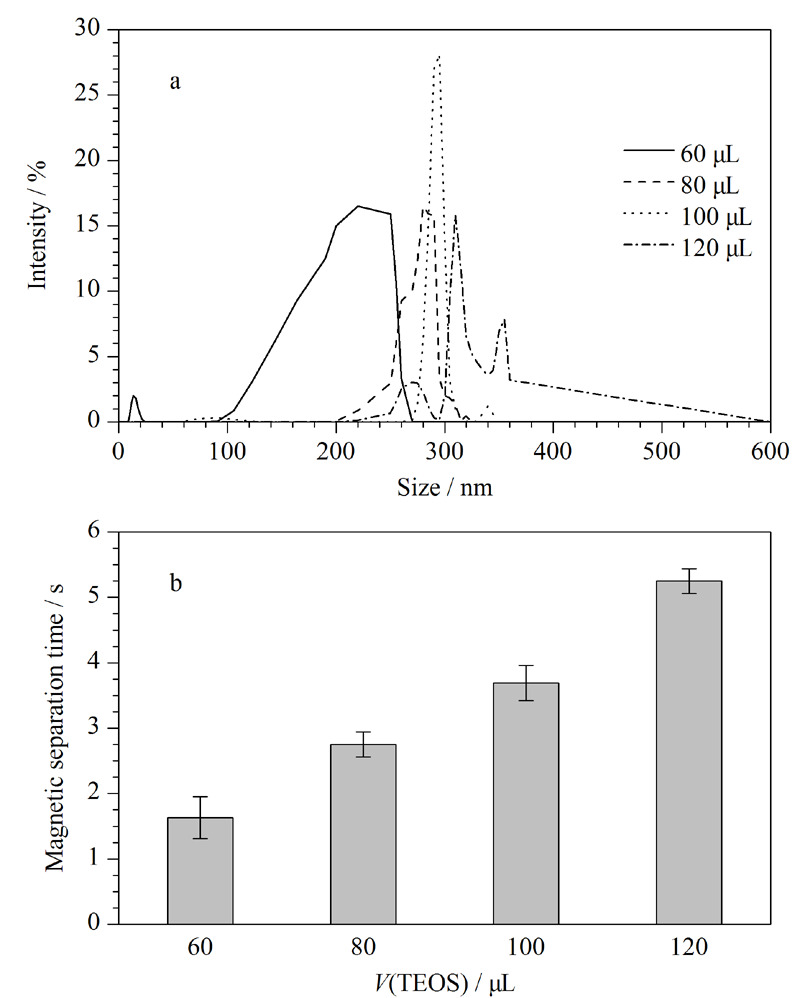
TEOS用量对(a)SiO_2_@Fe_3_O_4_粒径分布和 (b)磁分离时间的影响(*n*=3)

#### 2.1.2 抗体偶联条件优化

抗体的偶联是最为关键的一步,本实验通过BCA蛋白浓度测定试剂盒对SiO_2_@Fe_3_O_4_捕获的抗体量进行测定,分别对偶联过程中EDC与NHS物质的量之比、MES的pH值、MES的浓度以及偶联时间进行优化。如图3a所示,Ab与SiO_2_@Fe_3_O_4_磁性纳米材料的偶联效率明显受到EDC与NHS物质的量之比的影响,当EDC∶NHS为2∶1时,偶联效率逐渐增加,即与SiO_2_@Fe_3_O_4_磁性纳米材料结合的抗体逐渐增加,偶联率可达到76.47%~90.58%。

**图3:**
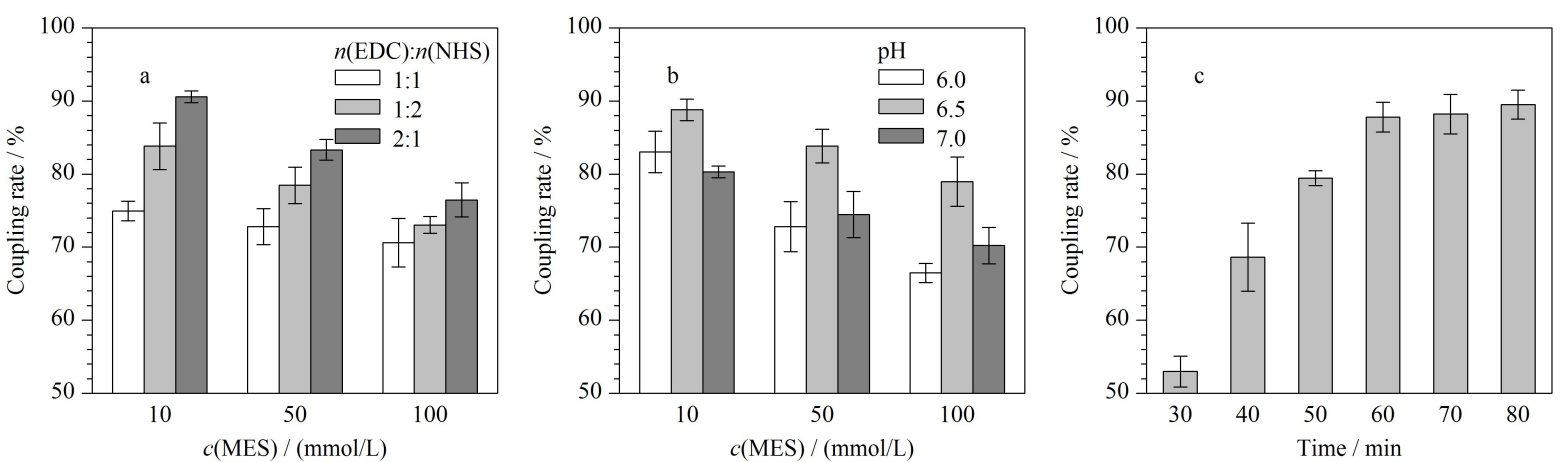
(a)EDC与NHS的物质的量之比、(b)MES的pH、浓度与(c)偶联时间对抗体偶联效率的影响(*n*=3)

这是由于抗体上的羧基在较多EDC存在的条件下可以形成不稳定的中间体,而NHS可以与该中间体结合形成稳定的活性酯附着在磁性纳米粒子表面,从而实现抗体的偶联^[18]^,如EDC占比过低,会对中间体的形成造成一定影响,导致抗体与SiO_2_@Fe_3_O_4_的偶联效率下降。随后比较了MES溶液不同pH值(6.0、6.5、7.0)以及不同MES浓度(10、50、100 mmol/L)对抗体偶联率的影响。结果表明,随着MES浓度的增加,偶联率逐渐降低,当采用pH=6.5、浓度为10 mmol/L的MES作为偶联缓冲液时表现出最佳偶联效果,偶联率可以达到88.79%(见图3b)。最后对抗体的偶联时间做了优化,如图3c所示,随着时间的延长,抗体在SiO_2_@Fe_3_O_4_上的偶联率逐渐增加,在60 min时增长变缓慢,Ab与SiO_2_@Fe_3_O_4_磁性纳米材料的结合趋于饱和,因此选取60 min作为最佳偶联时间。

### 2.2 SiO_2_@Fe_3_O_4_的表征

分别采用TEM、DLS、FT-IR对SiO_2_@Fe_3_O_4_的形貌、基团分布和粒径分布进行表征,证明材料的成功合成。由图4a可知,SiO_2_@Fe_3_O_4_的分散性较好,未发生团聚,粒径约为280 nm, SiO_2_包覆层的厚度约为25 nm。图4b为材料的粒径分布图,大约68.3%的Fe_3_O_4_磁性纳米颗粒的粒径分布在260 nm左右,所占比例较高,说明合成的Fe_3_O_4_磁性纳米颗粒分布较为均匀。约44.6%的SiO_2_@Fe_3_O_4_的粒径分布在275~285 nm之间,进一步说明了SiO_2_在Fe_3_O_4_磁性纳米颗粒上的成功包覆,包覆厚度大约为25 nm,与TEM的表征结果对应。图4c为Fe_3_O_4_磁性纳米颗粒、SiO_2_@Fe_3_O_4_和Ab-SiO_2_@Fe_3_O_4_的红外光谱图,SiO_2_@Fe_3_O_4_在1026 cm^-1^、1098 cm^-1^处出现了Si-O键的伸缩振动峰,Ab-SiO_2_@Fe_3_O_4_在1652 cm^-1^处出现了抗体的酰胺键的峰,属于酰胺Ⅰ带,说明SiO_2_在Fe_3_O_4_磁性纳米颗粒上的成功包覆和抗体的成功偶联,成功合成了Ab-SiO_2_@Fe_3_O_4_用于后续AFB_1_的分离富集。

**图4:**
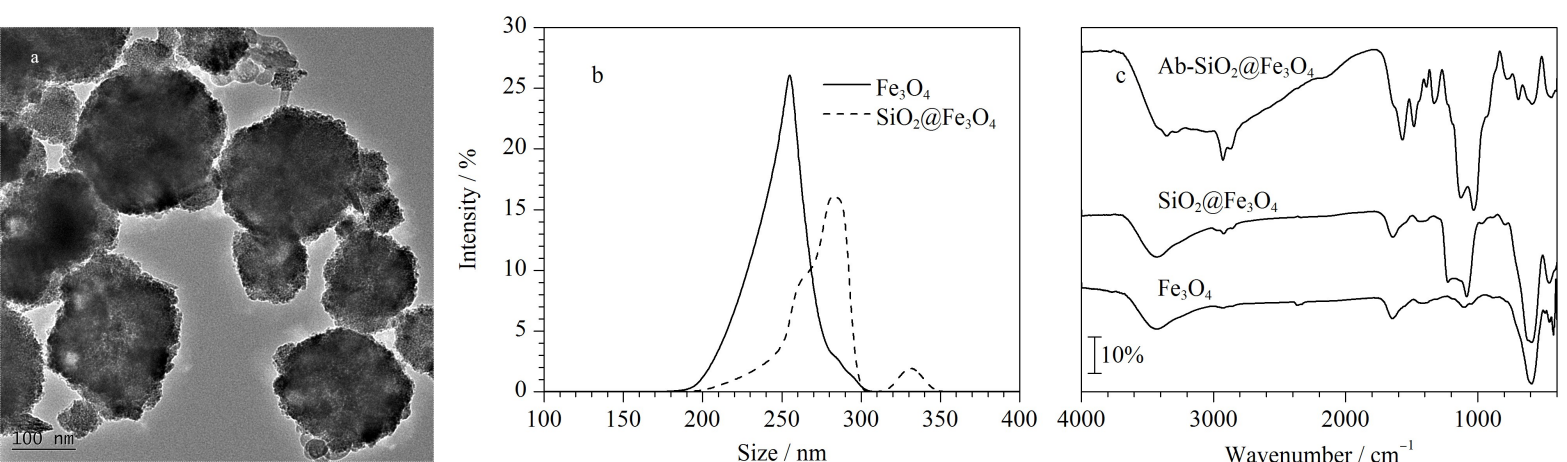
SiO_2_@Fe_3_O_4_的(a)透射电子显微镜、(b)动态光散射和(c)傅里叶变换红外光谱表征

### 2.3 Ab-SiO_2_@Fe_3_O_4_对AFB_1_分离富集条件优化

#### 2.3.1 吸附条件的优化

Ab-SiO_2_@Fe_3_O_4_对于AFB_1_的分离富集条件是影响材料应用效果的重要外部因素,本实验分别考察了所用PBS的pH、富集温度、Ab-SiO_2_@Fe_3_O_4_磁性纳米材料用量、富集时间对材料回收率的影响。取1 mL AFB_1_标准溶液(10 μg/mL,溶于pH=7.4的PBS),在不同条件下按照1.4节所述步骤进行AFB_1_的分离富集,采用HPLC-MS/MS测定回收率。如图5a所示,Ab-SiO_2_@Fe_3_O_4_对AFB_1_的回收率随着缓冲液pH值的增加出现先增加后减小的趋势,这主要归因于抗体与抗原在中性条件下具有较好的结合效率,因此后续采用pH=7.4的PBS溶液作为缓冲溶液。由图5b可知,富集温度为37 ℃时回收率最高,这由于37 ℃是免疫反应的最适温度^[19]^。图5c结果显示随着Ab-SiO_2_@Fe_3_O_4_磁性纳米材料用量的增加,AFB_1_溶液的回收率逐渐增加,当用量达到8 mg后,回收率不再发生明显变化,因此选择8 mg作为最佳Ab-SiO_2_@Fe_3_O_4_用量。图5d为达到富集平衡所需要的时间,富集10 min后回收率趋于平缓,表明已接近饱和状态,因此选择10 min作为富集时间。

**图5:**
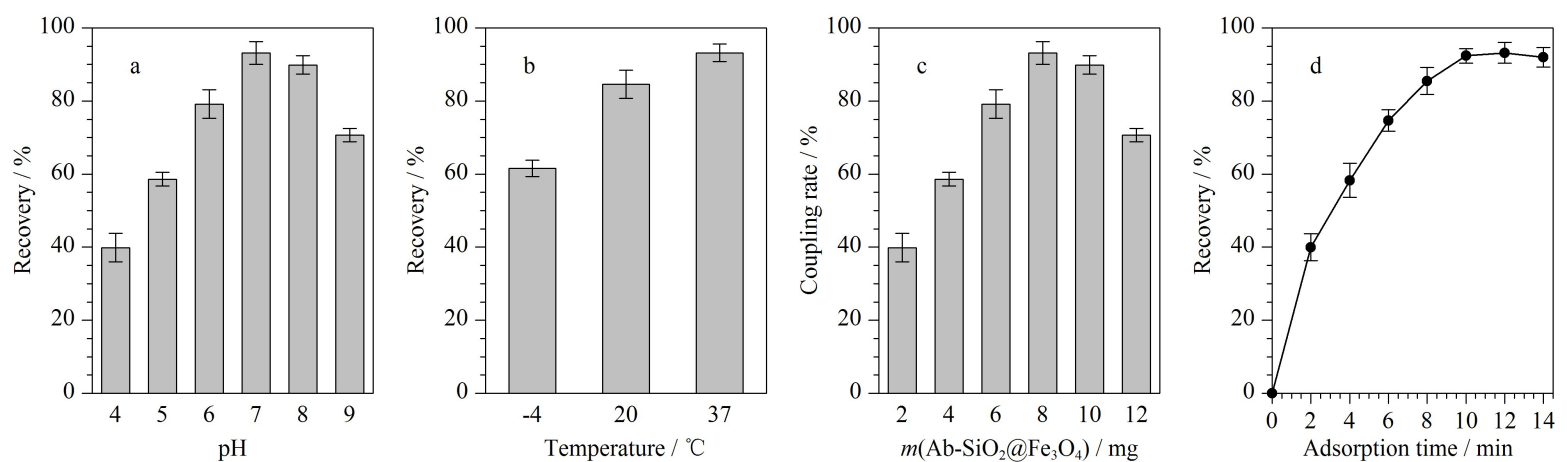
(a)缓冲液pH、(b)富集温度、(c)材料用量和(d)富集时间对AFB_1_回收率的影响(n=3)

#### 2.3.2 洗涤条件的优化

查阅文献^[16]^可知,Gly-HCl酸化液可促进抗原抗体复合物的解离,且添加一定浓度的去垢剂可进一步提升解离效果。因此选择在0.1 mol/L的Gly-HCl缓冲溶液中加入1%、2%、5%、10%的Tween-20或0.5%、1%、2%、5%的SDS,探究不同溶液对AFB_1_的洗涤效果。取1 mL AFB_1_标准溶液(10 μg/mL,溶于pH=7.4的PBS)在不同条件下按照1.4节所述步骤进行AFB_1_的分离富集,采用HPLC-MS/MS测定回收率。结果表明,在Gly-HCl缓冲溶液中添加2%的Tween-20时,可以达到最佳洗涤效果,回收率为96.57%,因此采用含有2%的Tween-20的Gly-HCl缓冲液将Ab-SiO_2_@Fe_3_O_4_分离富集的AFB_1_洗涤下来,进行后续试验。

### 2.4 Ab-SiO_2_@Fe_3_O_4_的性能验证

#### 2.4.1 分离富集能力验证

采用相同的偶联方法将AFB_1_抗体分别偶联到氨基化的裸Fe_3_O_4_磁性纳米颗粒和SiO_2_@Fe_3_O_4_磁性纳米材料上,合成了Ab-Fe_3_O_4_和Ab-SiO_2_@Fe_3_O_4_^[20]^。取1 mL AFB_1_标准溶液(10 μg/mL,溶于pH=7.4的PBS),分别采用Ab-Fe_3_O_4_和Ab-SiO_2_@Fe_3_O_4_按照1.4节所述方法进行分离富集,采用HPLC-MS/MS进行定量,做出两种材料对AFB_1_的富集曲线。如图6a所示,随着时间的增加,Ab-SiO_2_@Fe_3_O_4_相对于Ab-Fe_3_O_4_具有更高的回收率,且对AFB_1_具有更强的捕获能力。由公式(1)计算结果可知,在最佳的富集条件下,Ab-SiO_2_@Fe_3_O_4_磁性纳米材料对AFB_1_的最大富集容量为68.283 mg/g,这种高富集能力归因于该材料对AFB_1_抗体富集量的增加,导致单位质量材料中含有更多的AFB_1_结合位点^[21]^。

**图6:**
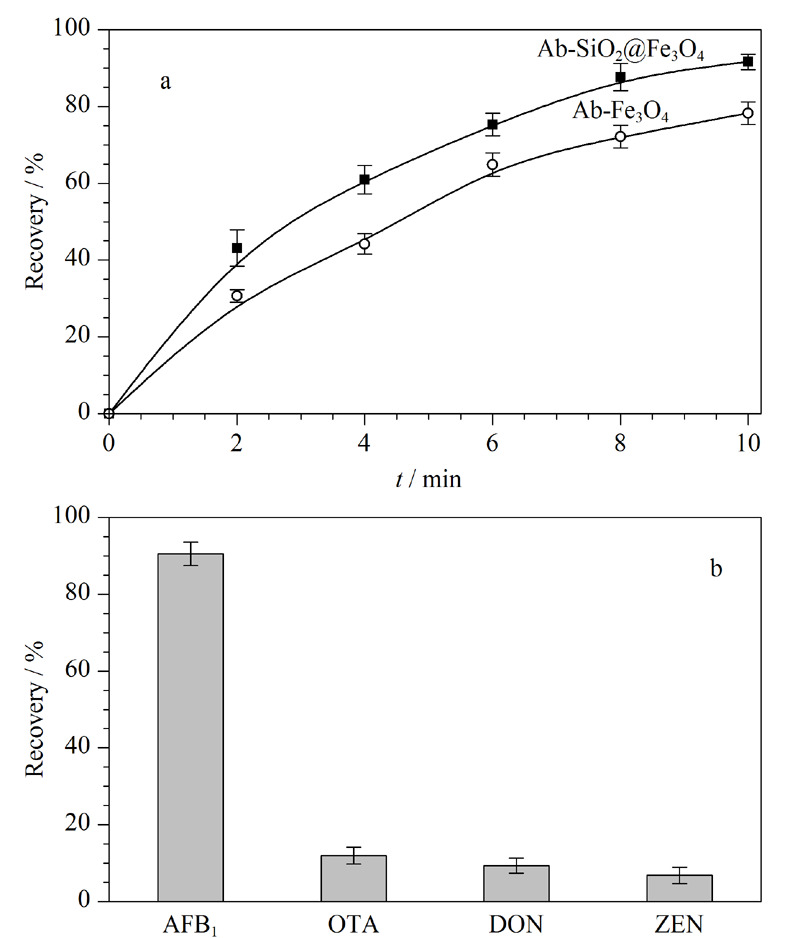
(a)Ab-SiO_2_@Fe_3_O_4_与Ab-Fe_3_O_4_的富集能力和(b)材料特异性(n=3)

选择OTA、DON、ZEN 3种真菌毒素作为干扰物来验证Ab-SiO_2_@Fe_3_O_4_磁性纳米材料对AFB_1_的特异性结合性能。如图6b表明,在相同的加标浓度下,该磁性纳米材料对AFB_1_的回收率大大高于其他3种真菌毒素,说明该材料对AFB_1_具有出色的选择性富集效果,说明免疫反应体系在分离富集过程的利用可以大大减少其他真菌毒素的干扰,实现目标物的准确检测。

为评价Ab-SiO_2_@Fe_3_O_4_磁性纳米材料在复杂谷物基质中的应用和重复使用性能,在5 g小麦样品提取液中分别加入0.1、0.5、1.0 ng/mLAFB_1_标准溶液,分离富集后,采用酶联免疫吸附法测定洗涤液中的AFB_1_含量,重复5次。结果表明,该材料在复杂样品中依然保持优异的富集能力,回收率为81.34%~89.96% (见表1),并且经多次使用后的Ab-SiO_2_@Fe_3_O_4_磁性纳米材料对AFB_1_的回收率变化不大,RSD≤4.96%,说明材料的重复使用性能良好。因此,Ab-SiO_2_@Fe_3_O_4_磁性纳米材料具有较好的分离富集能力和显著的特异性,在复杂的谷物基质中也有令人满意的表现。

**表1:** Ab-SiO_2_@Fe_3_O_4_磁性纳米材料在小麦样品中的 回收率和重复使用性

Repeat time	Spiked/ (μg/kg)	Detected/ (μg/kg)	Recovery/ %	RSD (n=5)/ %
1	0.1	0.086	85.86	4.36
	0.2	0.165	82.73	4.07
	0.5	0.446	89.26	3.95
2	0.1	0.094	83.95	2.98
	0.2	0.166	82.92	3.01
	0.5	0.444	88.73	3.62
3	0.1	0.087	86.82	4.18
	0.2	0.163	81.51	4.29
	0.5	0.450	89.96	4.32
4	0.1	0.085	84.57	3.15
	0.2	0.163	81.34	3.67
	0.5	0.448	89.61	4.96
5	0.1	0.085	84.61	3.75
	0.2	0.161	80.58	2.84
	0.5	0.4382	87.64	3.08

#### 2.4.2 储存性能验证

为验证Ab-SiO_2_@Fe_3_O_4_磁性纳米材料在4 ℃条件下的储存期限,分别在第1、3、5、7、9、11 d按照1.4节所述步骤对1 mL AFB_1_标准溶液(10 μg/mL,溶于pH=7.4的PBS)进行分离富集,探究储存不同天数后材料对AFB_1_的富集效果变化。最后采用1 mL PBS溶液(pH=8.0)对材料进行洗涤,从而保持材料表面抗体活性以供下次测定。如图7显示,第1~7 d内,材料的回收率有轻微波动,吸附效果整体处于较为稳定的水平,而在第9 d和11 d均出现明显的降低趋势,推测是Ab-SiO_2_@Fe_3_O_4_磁性纳米材料偶联的抗体活性随着时间的延长逐渐下降。因此,该材料可在至少7 d内保持良好的富集效果。

**图7:**
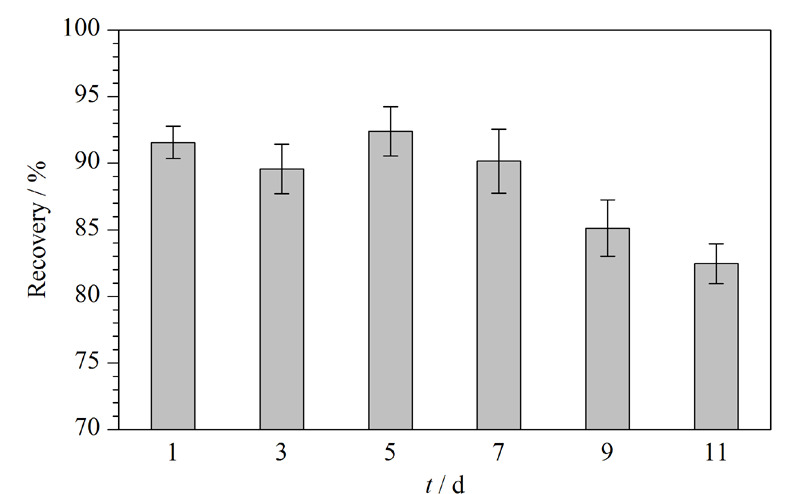
不同保存天数下Ab-SiO_2_@Fe_3_O_4_磁性纳米材料对AFB_1_的回收率(n=3)

### 2.5 谷物中AFB_1_的提取条件优化

在现有文献报道的基础上,研究了提取溶剂比例和提取条件对提取效果的影响^[22,23]^。本实验考察了不同比例的乙腈-水-甲酸对AFB_1_提取率的影响,通过高效液相色谱-串联质谱对添加AFB_1_标准品5 ng后的空白样品中提取出的AFB_1_进行定量检测来确定提取率。由图8可知,一定量的水可以促进AFB_1_在实际样品中的提取,而随着水的比例逐渐升高,提取液的提取效果逐渐减弱,推测是过量的水对甲酸产生了稀释作用,从而降低了甲酸对真菌毒素萃取效率的正向影响。另外,对比了颠倒混匀和超声两种方式的提取效果,颠倒混匀方法对谷物中AFB_1_的提取率为56.80%,而超声方法的提取率为92.63%,因此后续采用乙腈-水-甲酸(85∶10∶5, v/v/v)溶液作为提取剂,超声10 min的条件对样品中的AFB_1_进行提取。

**图8:**
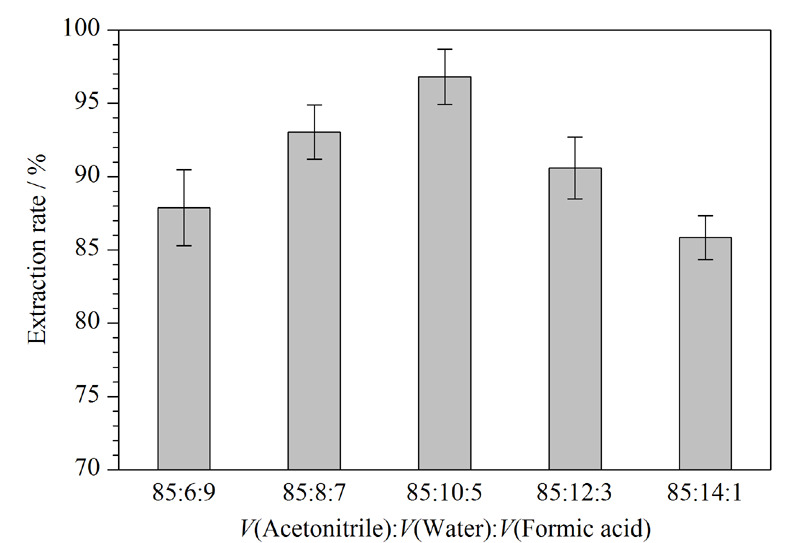
不同体积比的乙腈-水-甲酸对谷物中AFB_1_提取效率的影响

### 2.6 方法评价

#### 2.6.1 线性范围和检出限

将一定浓度梯度的标准AFB_1_溶液(0.1、1、2、5、10、20、40、50、60 μg/L)加入空白大米样品中,分离富集后进入高效液相色谱-串联质谱测定。以峰面积(*Y*)为纵坐标,AFB_1_的质量浓度(*X*, μg/L)为横坐标进行线性回归分析,AFB_1_在2~50 μg/L范围内具有良好的线性关系,相关系数(*R*^2^)=0.999。信噪比(*S/N*)分别为3和10时确定检出限(LOD)和定量限(LOQ),分别为0.04 μg/kg和0.13 μg/kg,远低于国家标准中规定的10 μg/kg;而采用国标GB 5009.22-2016第三法高效液相色谱-柱后衍生法在同样条件下的检出限和定量限分别为0.038 μg/kg和0.145 μg/kg,说明本方法提出的基于SiO_2_@Fe_3_O_4_的磁分离富集过程结合高效液相色谱-串联质谱法可以达到与标准方法相当的检测效果,且前处理方法更加简单高效,可满足实际样品中痕量检测的需求。

#### 2.6.2 回收率和精密度

为验证该方法在复杂谷物基质中检测的准确性和可靠性,选取3种常见谷物(大米、玉米和小麦)的空白样品,加入不同浓度的AFB_1_标准溶液,经1.3节提取和1.4节分离富集步骤后进样检测,计算加标回收率,每种水平重复测定5次。结果如表2所示,该方法的回收率范围为76.21%~92.85%, RSD≤5.29%。

**表2:** AFB_1_在3种谷物基质中4个水平下的加标回收率(*n*=5)

Food matrix	Spiked/ (μg/kg)	Detected/ (μg/kg)	Recovery/ %	RSD/%
Rice	0.1	0.089	88.75	4.88
	0.2	0.164	82.06	2.86
	0.5	0.393	78.57	3.16
	1.0	0.892	89.15	5.19
Corn	0.1	0.083	83.25	4.55
	0.2	0.152	76.21	3.28
	0.5	0.434	86.71	2.81
	1.0	0.818	79.45	4.94
Wheat	0.1	0.093	92.85	3.69
	0.2	0.172	86.03	5.29
	0.5	0.454	90.87	4.21
	1.0	0.886	88.56	3.95

#### 2.6.3 实际样品测定

从超市随机购买30份谷物样品,包含大米、玉米和小麦各10份,按照所建立的方法对该30份实际谷物样品中的AFB_1_进行检测。结果显示,在1个小麦样品和2个玉米样品中检出了AFB_1_,含量分别为0.38、0.13、0.47 μg/kg,均未超过国家标准所规定的限量指标,其余样品均未检出AFB_1_。采用GB 5009.22-2016第三法高效液相色谱-柱后衍生法对上述阳性样品中的AFB_1_含量进行复测,测得AFB_1_的含量分别为0.37、0.11、0.48 μg/kg,说明该方法具有较好的准确性。

基于SiO_2_@Fe_3_O_4_的磁分离富集过程结合高效液相色谱-串联质谱法与GB 5009.22-2016第三法高效液相色谱-柱后衍生法相比,优势主要体现在以下几个方面:一是检测快速,免疫亲和柱的净化过程需要2.5 h以上,而基于Ab-SiO_2_@Fe_3_O_4_的分离富集过程在30 min内即可快速完成,可节省80%以上的时间;二是操作简单方便,利用材料的磁性可以与复杂基质快速分离,无需多次洗脱和其他繁杂操作;三是成本较低,Ab-SiO_2_@Fe_3_O_4_的合成原料成本低,合成条件较为温和,且对AFB_1_具有较强的特异性吸附能力,可达到良好的分离富集效果,而免疫亲和柱价格昂贵,难以满足大批量样品的检测需求;四是结果更加准确,采用Ab-SiO_2_@Fe_3_O_4_不仅可以达到较高的富集效率,实现更加充分的富集过程,且与HPLC-MS/MS结合可以达到较高的准确性,可实现谷物样品中痕量AFB_1_的高效分析。

## 3 结论

本文采用微波辅助水热法合成了Fe_3_O_4_磁性纳米颗粒,进一步合成了Ab-SiO_2_@Fe_3_O_4_,采用该材料对3种谷物样品中的AFB_1_进行分离富集,结合HPLC-MS/MS定量检测,建立了一种高效检测AFB_1_的方法。该方法将基于免疫反应的高效分离富集材料Ab-SiO_2_@Fe_3_O_4_与灵敏度高的HPLC-MS/MS结合起来,线性关系、检出限和重复性等方法学指标均能满足不同谷物中AFB_1_的实际测定,且材料合成过程简单、成本低廉,前处理过程简单快速,检测过程高效准确,可满足大批量检测要求,对于谷物安全监管、真菌毒素风险评估和守护人类健康具有重要意义。
